# Characterization and Biological Characteristics of *Alternaria*, *Botryosphaeria*, *Pestalotiopsis*, and *Trichothecium* Species Associated with Postharvest Loquat Fruit Rot in Yunnan, China

**DOI:** 10.3390/plants14203201

**Published:** 2025-10-18

**Authors:** Jian-Wei Guo, Chun-Lian Yang, Beng-Zha Dong, Rong-Chuan Tian, Min Yang, Lifang Li, Penghua Gao, Su-Yue Zhou, Murad Muhammad, Yu Bu, Junbo Zhang, Chui-Si Kong, Lei Yu

**Affiliations:** 1 College of Agronomy and Life Sciences/Yunnan Urban Agricultural Engineering and Technological Research Center, Kunming University, Kunming 650214, China; gjwkf475001@sina.com (J.-W.G.); 15894424392@163.com (C.-L.Y.); 15108609703@163.com (B.-Z.D.); trww2017@163.com (R.-C.T.); yangmin7799@163.com (M.Y.); csqsmile@126.com (L.L.); gaopenghua8878@dingtalk.com (P.G.); suyuezhou@outlook.com (S.-Y.Z.); buyu0851@163.com (Y.B.); 2 State Key Laboratory of Desert and Oasis Ecology, Key Laboratory of Ecological Safety and Sustainable Development in Arid Lands, Xinjiang Institute of Ecology and Geography, Chinese Academy of Sciences, Urumqi 830011, China; muradbotany1@uop.edu.pk; 3 University of Chinese Academy of Sciences, Beijing 100049, China; 4 Kunming Edible Fungi Institute of All China Federation of Supply and Marketing Cooperatives, Kunming 650221, China; 15170211710@163.com; 5 Agricultural Environment and Resources, Yunnan Academy of Agriculture Sciences, Kunming 650200, China

**Keywords:** *Alternaria alternata*, *Botryosphaeria dothidea*, *Pestalotiopsis kenyana*, *Trichothecium roseum*, *Eriobotrya japonica*, multilocus phylogeny, disease index

## Abstract

Postharvest diseases caused by various fungal pathogens pose a significant threat to fruit quality, storage, and market value, making their identification and biological characterization essential for effective management strategies. This study examines the morphological and phylogenetic characteristics of *Alternaria*, *Botryosphaeria*, *Pestalotiopsis*, and *Trichothecium* species associated with loquat fruit rot in Yunnan, China. In May 2023, fruit rot of loquat in Yunnan, China, was classified into four types: ring rot, brown spot, black spot, and soft rot, with incidence rates of 4%, 6%, 6%, and 12%, respectively. Based on morphological features and molecular approaches, two strains of *Botryosphaeria* were identified as *Botryosphaeria dothidea*, which causes ring rot. Three strains of *Trichothecium* were identified as *Trichothecium roseum*, which is responsible for the brown spots. Three strains of *Alternaria* were identified as *Alternaria alternata*, which led to the appearance of black spots on the leaves. Similarly, two strains of *Pestalotiopsis* were identified as *Pestalotiopsis kenyana*, which causes soft rot. All identified species were verified to induce harvest loquat fruit rot by validating Koch’s postulates. This is the novel report of *B. dothidea*, *T. roseum*, and *P. kenyana* inducing postharvest fruit rot on loquat in Yunnan, China, and globally. It is also the first evidence that *A. alternata* causes postharvest fruit rot and gray leaf spot on loquat in Yunnan, China. The virulence differed among species, even within isolates of the same species. Additionally, the effect of temperature on the pathogenicity of *A. alternata* on loquat leaves was more than humidity. These findings enhance our understanding of the fungal pathogens affecting loquat fruit in the study area and highlight the importance of effective management strategies to minimize fruit rot. Further research is needed to investigate the ecological impacts of these species and potential control measures in agricultural practices.

## 1. Introduction

Loquat (*Eriobotrya japonica* Lindl.) is an evergreen, subtropical fruit tree originating from China, which is the largest producer with an annual yield surpassing 1 million tons [1,2]. Due to its delicious fruits and leaves, it is utilized in traditional Chinese medicine (TCM) to treat coughs and other respiratory disorders. The loquat has been cultivated for over 2100 years in China and has spread to the United States, Japan, Turkey, Spain, Pakistan, India, Brazil, Greece, and Cyprus [3]. It is primarily planted in China, mainly in Fujian, Sichuan, Chongqing, Yunnan, Zhejiang, Guangdong, Jiangsu, and other regions [4]. Among them, the planting area and yield in Yunnan Province are ranked fifth in China.

In the long history of cherishing loquat trees, fungal fruit diseases have caused severe economic losses. Nowadays, there are 17 species belonging to 12 genera, including *Colletotrichum* [5,6,7,8,9,10], *Pestalotiopsis* [7,11], *Neopestalotiopsis* [5,12,13], *Alternaria* [14], *Botrytis* [5], *Diplodia* [5], *Penicillium* [5], *Rhizopus* [5,15], *Ceratobasidium* [16], *Geotrichum* [17], and *Diplocarpon* [18], which have been reported to cause postharvest fruit rot of loquat worldwide; On the contrary, there are 15 species belonging to nine genera, including *Colletotrichum* [19,20,21,22], *Pestalotiopsis* [23,24], *Alternaria* [25,26,27], *Fusicladium* [28], *Diplodia* [29,30], *Fusarium* [31,32], *Neofusicoccum* [33], *Diplocarpon* [18], and *Monilinia* [34], which have been reported to be the cause of loquat fruit rot before harvest globally (Table 1). Among them, *C. acutatum*, *P. eriobotryfolia*, and *A. tenuis* were reported to cause postharvest fruit rot of loquat in Fujian province, China [7]; *C. nymphaeae* was reported to cause postharvest fruit rot of loquat in Sichuan province, China [10]; *N. parvum* was reported to cause before-harvest fruit rot of loquat in Chongqing, China [33]; *C. scovillei* was reported to cause postharvest fruit rot of loquat in Zhejiang province, China [9]; *Ceratobasidium* sp. was reported to cause postharvest fruit rot of loquat in Guangdong province, China [16]; *C. acutatum* was reported to cause postharvest fruit rot of loquat in Jiangsu province, China [8]; *Alternaria* sp. [27], *C. nymphaeae*, and *C. eriobotryae* [21] were reported to cause before-harvest fruit rot of loquat in Taiwan province, China; *M. fructicola* was reported to cause before-harvest fruit rot of loquat in Hubei province, China [34]. However, it remains unclear how many fungal pathogens can destroy postharvest loquat fruit in Yunnan Province, the fifth-largest producer of loquat fruit in China.

Traditionally, anamorphic fungal species are identified by morphological traits including colonies on natural substrates or culture media, reproductive structures (shape, size, and septation of conidia, glomerospores, sporangiospores, zoospores, or chlamydospores), sporulation patterns (conidiomata or naked conidia), conidiogenesis (conidiophores and conidiogenous cells), and hosts [35,36]. However, these characteristics vary with their growth stage and with environmental conditions such as different media and temperature, host, or natural substrate [37]. In recent years, multi-locus phylogeny combined with morphological characteristics has been proposed for distinguishing fungal species [38]. For example, Summerbell et al. (2011) proposed the application of the LSU to differentiate *Trichothecium* species [39], Woudenberg et al. (2013) proposed the application of a combination of the GAPDH, RPB2, and TEF1-α to differentiate *Alternaria* species [40], the multi-loci phylogeny of the ITS-, TUB-, and TEF-combined conidial characteristics was proposed to distinguish *Pestalotiopsis* species [41], and the phylogeny of the ITS and TEF1-α was proposed to distinguish *Botryosphaeria* species [42]. Hence, in the present study, fungal pathogens associated with symptomatic loquat fruits from Yongshan County, Zhaotong City, Yunnan Province, China, were identified using both morphological and multi-loci phylogeny, which is a fundamental task for the quick diagnosis, prevention, and control of postharvest loquat fruit rot.

## 2. Results

### 2.1. Natural Symptoms, Fungal Isolation

**Figure 1:**
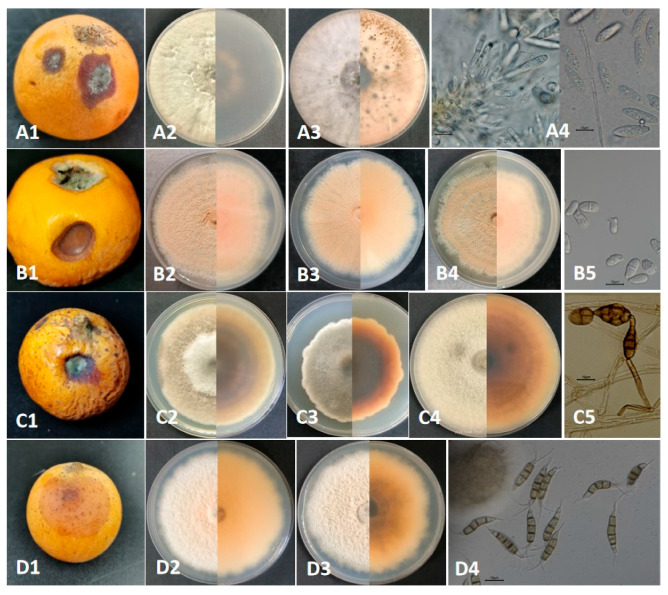
The naturally infected symptoms, colony features, and conidial characteristics of the *Botryosphaeria*, *Trichothecium*, *Alternaria*, and *Pestalotiopsis* species isolated from loquat fruit rot. (**A1**–**A4**) correspond to *Botryosphaeria dothidea*; (**B1**–**B5**) correspond to *Trichothecium roseum*; (**C1**–**C5**) correspond to *Alternaria alternata*; (**D1**–**D4**) correspond to *Pestalotiopsis kenyana*. Bars = 10 μm.

The disease symptoms and incidence rates of 200 loquat fruits harvested over two weeks were investigated in Yongshan County, Zhaotong City, Yunnan Province, China. The disease symptoms appeared two weeks after storage of loquat fruit at 14–30 °C with a relative humidity of 43–85%. Four types of diseases are involved in ring rot, brown spot, black spot, and soft rot, with incidence rates of 4%, 6%, 6%, and 12%, respectively (Figure 1). Early symptoms of ring rot on fruits appeared as dark brown ring spots, and later, the spots slowly coalesced to form large, irregularly marginated necrotic areas with gray mold at the center. However, the necrotic lesions did not become soft. The symptoms of the black spot appeared to be like those of the brown spot, initially a brown, round spot that gradually became significantly larger underwater. Finally, the disease spot of the black spot turned black and produced grayish-white mold at the center, while that of the brown spot remained bright brown. The symptom of soft rot appeared as a light brown, round spot, which subsequently became larger and softer.

A total of ten strains were isolated from symptomatic postharvest loquat fruits in Yongshan County, Zhaotong City, Yunnan Province, China. The ten strains were classed into four groups based on colony and conidial characteristics. The first group contained two strains, for instance, GJW41-13 and GJW42-17, isolated from ring rot; the second group contained three strains, GJW43-2, GJW43-10, and GJW43-17, from brown spot; the third group contained three strains, namely GJW44-5, GJW44-16, and GJW46-2, from black spot; and the fourth group contained two strains, namely GJW67-9 and GJW67-12, from soft rot.

### 2.2. Morphological and Molecular Identification of Potential Pathogenic Fungi

Colonies of GJW41-13 and GJW42-17 on PDA medium initially appeared white, with approximately circular or irregular margins at 27 °C. After three days of inoculation, the colonies GJW41-13 and 42-17 had diameters of 8.00cm and 9.00cm, respectively, and gradually became dark gray, with dense aerial mycelium. Their conidia were hyaline, subcylindrical, aseptate, and 17.5–24.1 × 5.7 to 7.2 μm, L/W = 2.7–4.1 (mean 21.0 × 6.6 μm, average L/W = 3.2, *n* = 30), like *Botryosphaeria dothidea* [(20-)23-27(-30) × 4-5(-6) (mean 26.2 × 5.4 μm)] [43]. Colonies of GJW43-2, GJW43-10, and GJW43-17 on PDA medium appeared light orange-red, with an orange powder and an irregularly rounded margin at 27 °C. After three days of inoculation, their colony diameters were 2.85 cm, 2.91 cm, and 2.54 cm, respectively. Their conidia were clusters, smooth, hyaline, thick-walled, ellipsoid to pyriform, 9.1–16.3 × 4.5–8.8 μm, L/W = 1.4–2.6 (mean 12.8 × 6.3 μm, average L/W = 2.1, *n* = 30), which was similar to *Trichothecium roseum* (10 to 18 × 7 to 9.5 μm) [44]. Colonies of GJW44-5, GJW44-16, and GJW46-2 on PDA medium appeared grayish-brown, white to gray with a flocculent texture on the front side, and a dark gray underside at 27 °C. After 3 days of inoculation, their colony diameters were 3.36 cm, 2.79 cm, and 3.58 cm, respectively. Their conidia were yellow-brown or black-brown, obclavate, subglobose, or ellipsoid, with 1–5 transverse septa and 1–3 longitudinal septa, 13.4–42.9 × 7.7–22.0 µm, L/W = 1.5–4.6 (mean 27.6 × 13.5 µm, average L/W = 2.8, *n* = 30), chain-typed, produced singly, separated, and pale brown conidiophores, which were similar to *Alternaria alternata* [26–30 × 5–9 µm with 4–7 transverse septa and a few or no longisepta] [45]. Colonies of GJW67-9 and GJW67-12 on the PDA medium were white and pale yellow on the reverse side at 27 °C. After three days of inoculation, the colony diameters were 3.33 cm and 3.28 cm, respectively. Pycnidia were black and scattered on the mycelial mats after 15–20 d. Conidia were fusoid, ellipsoid, straight or slightly curved, 4-septate, 20.9–27.6 × 4.0–5.4 µm, L/W = 4.5–6.9 (mean 24.3 × 4.6 µm, average L/W = 5.5, *n* = 20); three median cells were darker than other cells; the apical cell with 2–3 tubular appendages (mainly three) ranged from 6.6 to 16.3 µm, and the basal cell had one tubular appendage, ranging from 4.1 to 7.6 µm, which was similar to *Pestalotiopsis kenyana* [17.5–22 × 6–7 µm (mean 20 × 6.5 µm)] (Table 2) [46].

**Figure 2:**
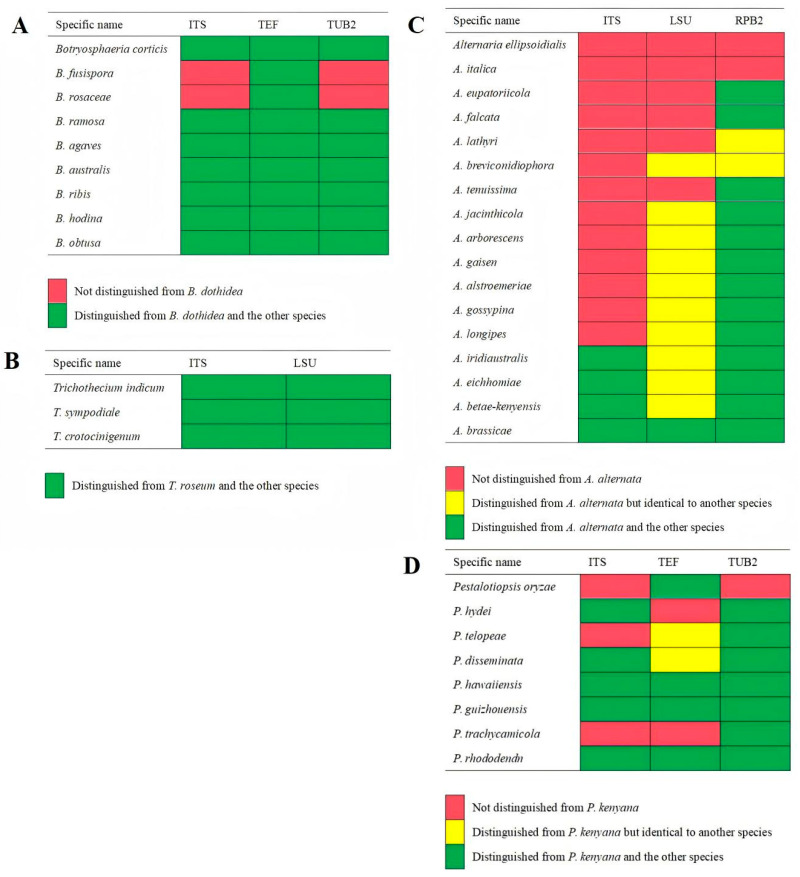
Comparison of gene ability to distinguish among different species. (**A**) Comparison of gene ability to distinguish *Botryosphaeria dothidea* from other *Botryosphaeria* species, (**B**) comparison of gene ability to distinguish *Trichothecium roseum* from other *Trichothecium* species, (**C**) comparison of gene ability to distinguish *Alternaria alternata* from other *Alternaria* species, (**D**) comparison of gene ability to distinguish *Pestalotiopsis kenyana* from other *Pestalotiopsis* species.

**Figure 3:**
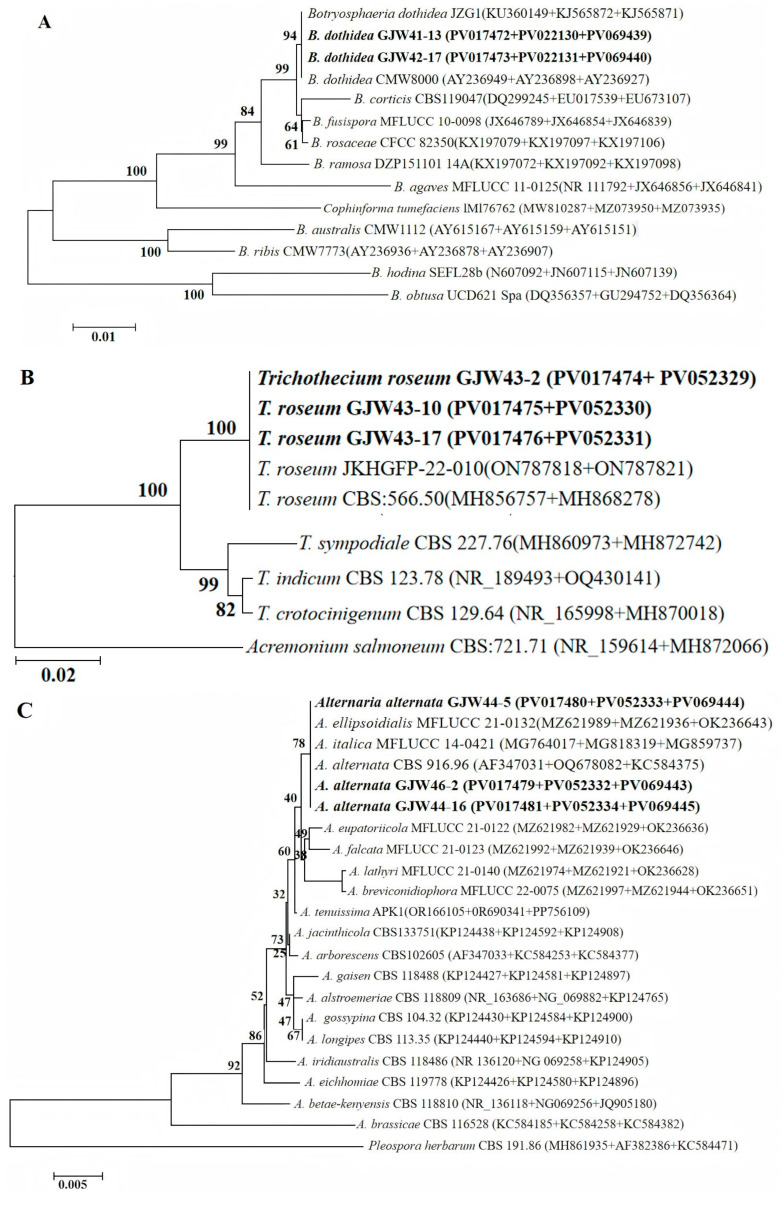

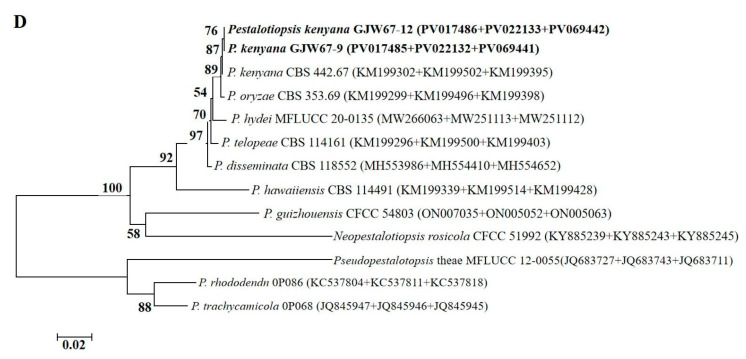
N-J phylogenetic trees of *Botryosphaeria dothidea*, *Trichothecium roseum*, *Alternaria alternata,* and *Pestalotiopsis kenyana* causing postharvest ring rot, brown spot, black spot, and soft rot of loquat in Yunnan, China. (**A**) *B. dothidea* based on ITS-, TEF-, and TUB2-sequenced data, (**B**) *T. roseum* based on ITS- and LSU-sequenced data, (**C**) *A. alternata* based on ITS-, LSU-, and RPB2-sequenced data, and (**D**) *P. kenyana* based on ITS-, TEF-, and TUB2-sequenced data. The strains from this study are indicated in bold. The scale bar shows the number of expected changes per site.

Preliminary morphological analysis of all the strains revealed that those belonging to groups I to IV were similar to *B. dothidea*, *T. roseum*, *A. alternata*, and *P. kenyana*, respectively. Thus, the strains from each morphotype were selected to confirm the classification results through single or multiple loci phylogenetic analyses. Firstly, the single-gene phylogenies are approximately consistent with multiple loci phylogenies; however, some of the genes sequenced in this study do not enable us to distinguish all of the species recognized here on their own, especially for the single-gene phylogenies of ITS within the genus *Alternaria* (Figure 2). Specially, the single-gene phylogenies of ITS and TUB2 cannot distinguish *B. dothidea* from *B. fusispora* and *B. rosaceae*; those of ITS and LSU fully distinguish *T. roseum* from *T. indicum*, *T. sympodiale*, and *T. crotocinigenum*; the ITS phylogeny cannot distinguish *A. anternata* from *A. ellipsoidialis*, *A. italica*, *A. eupatoriicola*, *A. falcata*, *A. lathyri*, *A. breviconidiophora*, *A. tenuissima*, *A. jacinthicola*, *A. arborescens*, *A. gaisen*, *A. alstroemeriae*, *A. gossypina*, and *A. longipes*; the LSU phylogeny cannot distinguish *A. anternata* from *A. ellipsoidialis*, *A. italica*, *A. eupatoriicola*, *A. falcata*, *A. lathyri*, and *A. tenuissima*; and neither can distinguish *A. alstroemeriae* from *A. breviconidiophora*, *A. jacinthicola*, *A. arborescens*, and *A. gaisen*, *A. gossypina* from *A. longipes*, *A. iridiaustralis* from *A. eichhomiae*, and *A. betae-kenyensis*; in contrast to that, the RPB2 phylogeny cannot distinguish *A. alternata* from *A. ellipsoidialis* and *A. italica*; neither can distinguish *A. lathyri* from *A. breviconidiophora*; the ITS phylogeny cannot distinguish *P. kenyana* from *P. oryzae*, *P. telopeae* and *P. trachycamicola*, and the TEF phylogeny cannot distinguish *P. kenyana* from *P. hydei*, nor can it distinguish *P. telopeae* from *P. disseminate*; in contrast that, the TUB2 phylogeny only cannot distinguish *P. kenyana* from *P. oryzae*. Secondly, the aligned concatenated sequences of 11 strains of *Botryosphaeria*, including 2 strains from loquat in the present study, and one strain of *Cophinforma tumefaciens* as the out-group, contained 1035 characters, including gaps (ITS: 1–430; TEF: 431–670; TUB2: 671–1035). For *Trichothecium*, the aligned combined sequences of five reference strains, three from this study and one strain of *Acremonium salmoneum* as the outgroup, comprised 917 characters, including gaps (ITS: 1–479; LSU: 480–917). For *Alternaria*, multigene sequence alignment of 18 reference strains, 2 strains from this study, and 1 strain of *Pleospora herbarum* as an outgroup included 1300 characters, including gaps (ITS: 1–547; LSU: 548–1019; RPB2: 1020–1300). For *Pestalotiopsis*, multigene sequence alignment of nine reference strains, two strains from this study, one strain of *Pseudopestalotopsis theae,* and one strain of *Neopestalotiopsis rosicola* as an outgroup included 1031 characters, including gaps (ITS: 1–496; TEF: 497–738; TUB2: 739–1031). The phylogram of *Botryosphaeria* shows that strains GJW41-13 and GJW42-17 (Group I) clustered with *B. dothidea* (JZG1 and CMW8000); strains GJW43-2, GJW43-10, and GJW43-17 (Group II) clustered with *T. roseum* (JKHGFP-22-010, CBS: 566.50); GJW44-5, GJW44-16, and GJW46-2 clustered with *A. alternata* (CBS 916.96), *A. ellipsoidialis* (MFLUCC 21-0132), and *A. italica* (MFLUCC 14-0421); and GJW67-9 and GJW67-12 (Group Ⅳ) clustered with *P. kenyana* (CBS 442.67) (Figure 3).

### 2.3. Pathogenicity on Loquat Fruits

**Figure 4:**
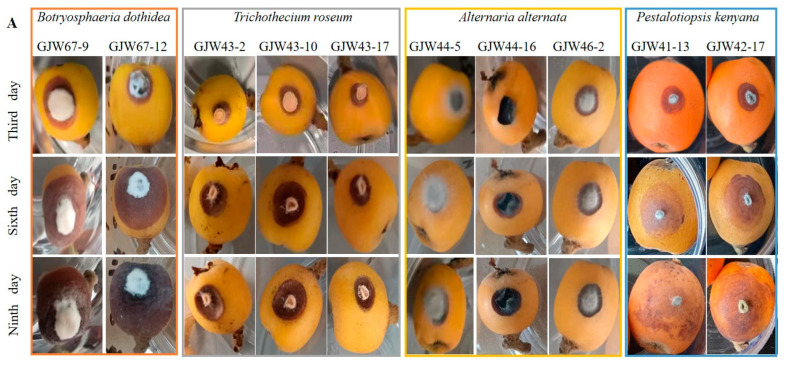

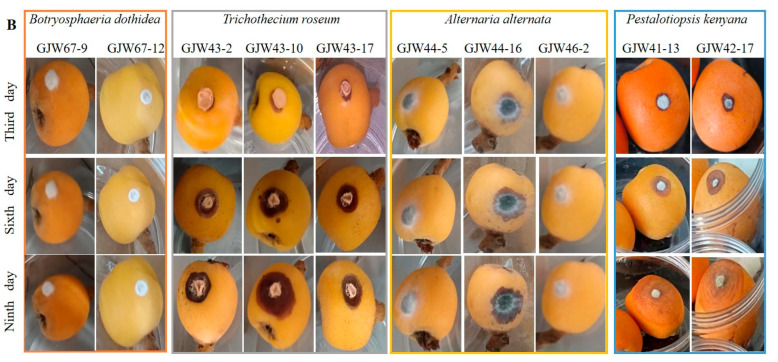
Artificially infected fruits of loquat with wounded inoculation and non-wounded inoculation, with mycelial plug. (**A**) The symptoms of artificially infected fruits with wounded inoculation with the mycelial plugs of *Botryosphaeria dothidea* GJW67-9 and GJW67-12, *Trichothecium roseum* GJW43-2, GJW43-10, and GJW43-17, *Alternaria alternata* GJW44-5, GJW44-16, and GJW46-2, *Pestalotiopsis kenyana* GJW41-13 and GJW42-17, respectively. (**B**) The symptoms of artificially infected, non-wounded inoculated fruits with the mycelial plugs of *B. dothidea* GJW67-9 and GJW67-12, *T. roseum* GJW43-2, GJW43-10, and GJW43-17, *A. alternata* GJW44-5, GJW44-16, and GJW46-2, *P. kenyana* GJW41-13 and GJW42-17, respectively. All treatments were maintained at 27 °C with a relative humidity of 60% under normal light conditions.

**Figure 5:**
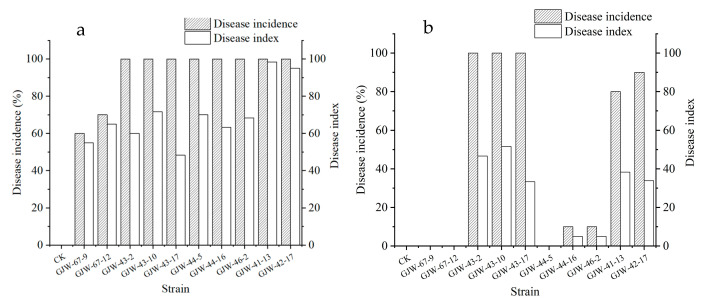
Incidence rate and disease index of postharvest fruit rot artificially infected with the mycelial plug of *Botryosphaeria dothidea* GJW67-9 and GJW67-12, *Trichothecium roseum* GJW43-2, GJW43-10, and GJW43-17, *Alternaria alternata* GJW44-5, GJW44-16, and GJW46-2, *Pestalotiopsis kenyana* GJW41-13 and GJW42-17, respectively. (**a**) All the treatments were inoculated on wounded sites; (**b**) all were inoculated on non-wounded sites.

The pathogenicity tests on the loquat fruits showed that all the strains caused symptoms at the wounded site after two days of inoculation. Among them, GJW43-2, GJW43-10, GJW43-17 (*T. roseum*), GJE44-5, GJW44-16, GJW46-2 (*A. alternata*), and GJW41-13 and GJW42-17 (*B. dothidea*) produced symptoms on fruits at both the needle-wounded and non-wounded sites (Figure 4); however, the lesions, incidence rate, and disease index on the needle-wounded sites were more prominent than on the non-wounded sites (Figure 5). All the infected fruits developed symptoms similar to those observed in the market and stockroom. No symptoms appeared on either the control needle-wounded fruits or the non-wounded fruits. Moreover, the fungi were re-isolated from the inoculated fruits. They exhibited colonial and conidial features similar to those of the original strains, confirming that the ten strains in this study cause loquat ring rot, brown spot, black spot, and soft rot, respectively.

**Figure 6:**
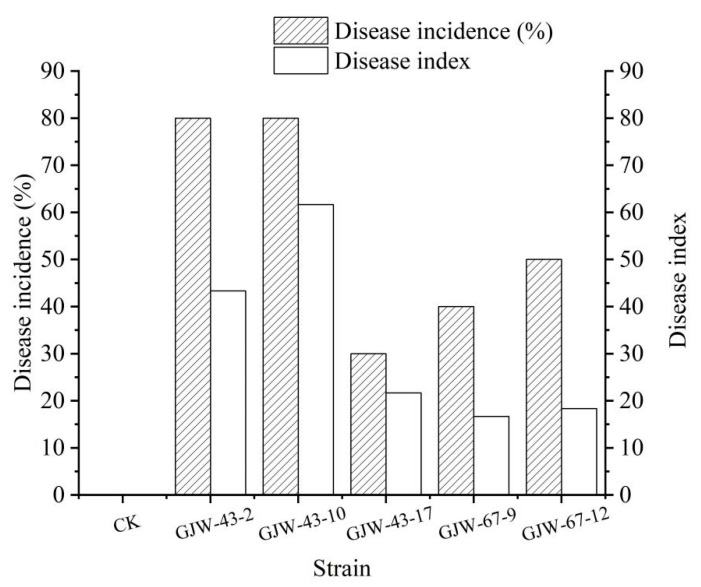
Incidence rate and disease index of postharvest non-wounded fruit rot artificially infected with a conidial suspension of five strains of *Botryosphaeria dothidea* GJW67-9 and GJW67-12, and *Trichothecium roseum* GJW43-2, GJW43-10, and GJW43-17, respectively.

After nine days of incubation at 27 °C at a relative humidity of 60% in the light, disease incidence and disease index (severity) on loquats wound-inoculated with *B. dothidea* were 100% and 95–98.33, *A. alternata* 100% and 63.33–70, *T. roseum* 100% and 48.33–71.67, and *P. kenyana* 60–70% and 55.00–65.00 (Figure 6). After 9 days in the same conditions, these values on intact-inoculated loquats were as follows: *B. dothidea* 0, *A. alternata* 0–10% and 0–50, *T. roseum* 100% and 33.33–51.67, and *P. kenyana* 80–90% and 33.87–38.33 (Figure 6). In summary, the virulence of *T. roseum* and *P. kenyana* was more potent than that of *B. dothidea* and *A. alternata*. Additionally, the virulence of four pathogens varied, even though they belonged to the same species.

After nine days of inoculation at 27 °C at a relative humidity of 60% in the light, the disease incidence rate and disease index (severity) on loquats sprayed with the conidial suspension of *B. dothidea* were 40–50% and 16.67–18.33, respectively, and for *T. roseum,* they were 30–80% and 21.67–61.67, respectively. The virulence of *T. roseum* was more substantial than that of *B. dothidea*.

After 16 days of inoculation of whole plants with a 7 mm mycelial plug of *B. dothidea*, disease incidence on needle-wounded fruits by GJW41-13 and GJW42-17 was 90% and 85%, respectively. In contrast, the non-wounded fruits were 85% and 60%. The symptoms were similar to those of naturally infected fruits, but some diseased fruits were rotten.

### 2.4. Effect of Temperature and Humidity on Pathogenicity of Alternaria alternata on Loquat Leaves

After 5 days of inoculation at a relative humidity of 50% in the darkness, disease incidence rates on loquat leaves with mycelial plugs of GJW44-5, GJW44-16, and GJW46-2 at 15 °C, 25 °C, and 30 °C were 100%. However, the disease index (severity) of GJW44-5, GJW44-16, and GJW46-2 increased with increasing temperature, reaching 68, 70, and 82, respectively (Table 3).

Compared to temperature, the effect of humidity on pathogenicity was not noticeable. After 6 days of inoculation at 25 °C in darkness, disease incidence rates on loquat leaves with mycelial plugs of GJW44-5, GJW44-16, and GJW44-16 at relative humidity levels of 45%, 55%, 65%, 75%, 85%, and 95% were 90–100%. The disease index (severity) of GJW44-5, GJW44-16, and GJW44-16 at a suitable humidity of 85% was 44 (Table 4).

## 3. Discussion

Many studies have shown that a complex infection of multiple pathogens usually causes disease, and the dominant pathogens in different regions may vary [47,48,49,50]. Therefore, it is crucial to implement effective management strategies to identify symptoms and respond to pathogens accurately. For example, 38 out of 195 diseases of significant grain and oil crops in China are caused by at least two fungal pathogens; among them, 9 *Fusarium* species caused maize seedling blight, 15 *Fusarium* species caused maize root rot, and maize basal rot was caused by 18 species of *Fusarium* [47,48,49], 338 strains from peach brown rot in Beijing are *M. fructicola*, and 4 strains are *M. fructigena*, which indicated that the dominant pathogen of peach brown rot in Beijing is *M. fructicola* [50]. Morphological characteristics are essential but insufficient for the classification of fungal species at the level of the genus, so strains from postharvest loquat were preliminarily grouped based on colony and conidial morphology. They were identified by multilocus phylogenetic analysis. Seven strains were identified as *B. dothidea*, *T. roseum,* and *P. kenyana*. Of them, *B. dothidea* has been reported to be associated with pre-harvest or postharvest fruit rot of Chinese olive, plum, pomegranate, yellowhorn, jujube, and apple in China [51,52,53,54,55,56], *T. roseum* has been reported to be associated with postharvest fruit rot of purple passion, orange, tomato, apple, strawberry, Hami melon, peach (*Prunus davidiana*), and peppers (*Capsicum* spp.) [57,58], *P. kenyana* has been reported to be the agent of bayberry leaf blight in Zhejiang Province, leaf spot on *Zanthoxylum schinifolium* in Sichuan, and *Rhododendron agastum* in Guizhou, China [59,60,61]. The other three strains were morphologically similar to *A. alternata* but clustered with *A. alternata*, *A. ellipsoidialis*, and *A. italica*. In contrast with the size of *A. alternata* (13.4–42.9 × 7.7–22.0 µm), that of *A. ellipsoidialis* was 45–70 × 15–30 μm, and that of *A. italica* was 76–98 × 23–39 μm. Therefore, GJW44-5, GJW44-16, and GJW46-2 were identified as *Alternaria alternata* based on morphological characteristics and phylogenetic analyses. *A. alternata* has previously been reported as the causal agent of gray spots on pre-harvest loquat fruit in Greece and Palestine, as well as postharvest loquat fruit rot in Pakistan [14,25,26]. To the best of our knowledge, this is the first report of *B. dothidea*, *T. roseum*, and *P. kenyana* causing postharvest fruit rot on loquat in Yunnan, China, as well as worldwide. Additionally, this is the first report of *A. alternata* causing postharvest fruit rot on loquat in Yunnan, China.

On the basis of comparison of the single-loci phylogenies combined with the multi-loci phylogenies, ITS and LSU are proposed for the application of distinguishing different *Trichothecium* species; TEF should be applied prior to the application for distinguishing different *Botryosphaeria* species. The multi-loci phylogenies combined with morphological characteristics must be used to distinguish different *Alternaria* and *Pestalotiopsis* species, as none of the genes sequenced in this study can enable us to distinguish them.

The pathogenicity test on loquat fruits verified that all species produced symptoms at the wounded sites, and *B. dothidea*, *T. roseum*, and *A. alternata* produced symptoms on fruits at the intact sites by inoculation with a 7 mm mycelial plug. However, *P. kenyana* could produce symptoms on fruits at non-wounded sites by inoculation with conidial suspension, and *B. dothidea* could cause symptoms of fruit-handing on loquat trees. This finding indicates that wounding is conducive to infection and colonization by these strains. Prior research has shown that injury enhances the infectivity and pathogenicity of harmful fungi [22,62]. In addition, the virulence of *T. roseum* and *P. kenyana* was more potent than that of *B. dothidea* and *A. alternata*, which is consistent with the pathogenicity tests of *Diplodia*, *Lasiodiplodia*, and *Neofusicoccum* species causing the *Botryosphaeria* canker and the dieback of apple trees in Central Chile [63]. The effect of temperature on the pathogenicity of *A. alternata* on loquat leaves was more than humidity. Many studies have documented that all strains were more virulent on wounded plant tissues than on non-wounded ones [64,65], and virulence is varied among species, even strains, temperature, and humidity [66,67,68]. Therefore, the loquat fruits should be gently plucked, packed, loaded, and unloaded during harvest/handling, as well as stored and transported under 15 °C to reduce commercial risks. In the future, the immunogold labeling technology of fruit rot diagnosis and fungicide sensitivity screening will be developed based on these four pathogens.

## 4. Materials and Methods

### 4.1. Field Sampling and Isolation

In May 2023, the incidence of postharvest loquat fruit rot was investigated in Yongshan County, Zhaotong City, Yunnan Province, China. Ten diseased fruits were collected and transported to the laboratory for fungal isolation within three days, during which time the disease symptoms were recorded.

The diseased fruits were surface-sterilized by immersing them in 75% ethanol for 30 s, followed by a 1 min exposure to 2.5% sodium hypochlorite, and then rinsed five times with sterilized distilled water. Subsequently, tissue pieces (5 × 5 mm) were cut from the edge of necrotic lesions. Finally, the tissue pieces were on sterilized filter paper for 3–5 min to dry them and transferred onto rose bengal agar (RBA; 5.00 g peptone, 10.00 g glucose, 1.00 g potassium dihydrogen phosphate, 0.50 g magnesium sulfate, 0.03g rose bengal sodium salt, 0.10 g chloromycetin, 15.00 g agar per L) in Petri dishes at 25 °C for three days. Hyphal tips were picked and transported to new RBA or potato dextrose agar (PDA; 200.00 g potatoes, 20.00 g glucose, 0.10 g chloromycetin, 15.00 g agar/L) plates and incubated in the dark at 25 °C.

### 4.2. Pathogenicity Test

Inoculation of detached mature fruits was performed with a 7 mm mycelial plug. Ten isolates, including three strains of *Alternaria* sp., two strains of *Botryosphaeria* sp., two strains of *Pestalotiopsis* sp., and three strains of *Trichothecium* sp., were used to inoculate detached mature fruits of the loquat cultivar Big Five-pointed Star. Healthy mature loquat fruits were harvested from the fields of Xiluodu town, Yongshan County, Zhaotong City, Yunnan Province, China. They were inoculated as previously described by Nozawa et al. [11] with some modifications. Fruits were washed with sterilized water, disinfected with 75% ethanol for 1 min, and then air-dried. A 7 mm mycelial plug from the colony edge was placed upside down on wounded or non-wounded fruit surfaces; another 7 mm agar plug was placed upside down on wounded or non-wounded fruit surfaces as a control. Ten wounded fruits and ten non-wounded fruits were used per strain. All treatments have been maintained at 27 °C with a relative humidity of 60% in the light. All treatments have been kept at 27 °C with a relative humidity of 60% under normal light conditions. According to Koch’s postulates, all fungal strains used in the pathogenicity tests were re-isolated from artificially infected fruits to ensure their identity, as described above.

Inoculation of detached mature fruits was performed with conidial suspension. A 10 µL drop of conidial suspension (1 × 10^6^ conidia/mL) by harvesting conidia from two-week-old PDA grown in the dark at 25 °C was sprayed on each non-wounded fruit, and another 10 µL drop of 0.05% Tween-80 was sprayed on each non-wounded fruit as a control [69]. Ten wounded fruits and ten non-wounded fruits were used per strain of *Pestalotiopsis* and *Trichothecium*. The environmental conditions and re-isolation of all fungal strains and loquat fruits tested were the same as described above.

Inoculation of whole plants was performed with a 7 mm mycelial plug. Twenty healthy fruits on one healthy plant with a stem base of 10 cm growing on the campus of Kunming University were inoculated separately with the 7 mm mycelial plug of each strain of Botryosphaeria, respectively. Twenty healthy fruits on another healthy plant were inoculated with a 7 mm agar plug as a control. Then, all fruits were individually covered with moist, sterile cotton and placed in transparent polyethylene bags for five days to maintain high relative humidity and an outdoor temperature of 13 to 24 °C.

The incidence rate (IR) and disease index (DI) were evaluated. IR (diseased fruits/total number of fruits inoculated) × 100%. They were referring to assessing the severity of brown leaf spots of Italian ryegrass [70] with modification. In brief, the degree of loquat fruit infection by the fungi was divided into seven grades according to disease scores ranging from 0 to 6 (0: healthy fruits; 1: <1% of the fruit surface shows water-soaked spots; 2: 1–<5% of the fruit surface shows water-soaked spots; 3: 5–<10% of the fruit surface shows brown or black spots; 4: 10–<30% of the fruit surface shows brown or black spots; 5: 30–<70% of the fruit surface shows brown or black spots; 6: >70% of the fruit surface shows brown or black spots, or the fruit falls off). The DI was calculated using the following formula: DI = (∑disease grade × number of diseased fruits)/(total inoculated fruits × 6).

IR and DI of *A. alternata* on loquat leaves were also used to assess the severity of brown leaf spots of Italian ryegrass [70] with modification. In brief, the degree of loquat leaf infection by the fungi was divided into seven grades according to disease scores ranging from 0 to 5 (0: healthy leaves; 1: <1% of the leaf surface shows diseased spots; 2: 1–<5% of the leaf surface shows diseased spots; 3: 5–<10% of the leaf surface shows diseased spots; 4: 10–<15% of the fruit surface shows diseased spots; 5: <15% of the leaf surface shows diseased spots). The DI was calculated using the following formula: DI = (∑disease grade × number of diseased fruits)/(total inoculated fruits × 5).

### 4.3. Morphological Identification and Characterization

For descriptions of colony appearance, the isolated strains were incubated on PDA plates for 14 d in the dark at 25 °C. The conidiogenous cells, from which conidia were formed on RBA or PDA of fungal strains, were photographed and measured using an Olympus BX53 microscope (Olympus, Tokyo, Japan). Conidia (*n* = 20 or 30) of each species were randomly selected for morphological analysis.

### 4.4. Genomic DNA Extraction, Sequencing, and Phylogenetic Analysis

The genomic DNA was extracted from the mycelium cultivated on PDA media plates at 25 °C for 5–7 days using a Solarbio Fungi Genomic DNA Extraction Kit (Beijing Solarbio Science & Technology Co., Ltd., Beijing, China) according to the manufacturer’s protocol. The internal transcribed spacer (ITS) regions were first amplified and sequenced using the primers ITS1/ITS4 [71] for all pathogenic strains. The strains were initially classified at the genus level as *Alternaria*, *Pestalotiopsis*, and *Trichothecium* based on conidial morphology. They were identified as *Botryosphaeria* by BLAST analysis of ITS sequences in the nucleotide database. The LSU gene region was amplified and sequenced for the *Alternaria* strains using the primers LR0R and LR5. In contrast, the partial RNA polymerase II second-largest subunit (RPB2) was amplified and sequenced using the primers RPB2-5f2/fRPB2-7CR [72]. Translation elongation factor (TEF) using primers EF1-728F/EF1-986R [59] and partial beta-tubulin gene (TUB2) using primers T1/Bt2b [73] were then amplified and sequenced for strains of *Botryosphaeria* and *Pestalotiopsis*. The LSU gene region, using primers LR0R and LR5 [72], was amplified and sequenced for the *Trichothecium* strain.

The PCR reaction volume was followed by Doilom et al. [74]. The PCR reaction primers and PCR parameters for each fungal genus are listed in Table 5. The amplified PCR products were forwarded to Tsingke Biotechnology Co., Ltd., Beijing, China, a commercial sequencing service, for sequencing.

The sequence information was uploaded to GenBank. Using MEGA7.0, the sequences were aligned. Gaps were interpreted as missing data, and ambiguous areas were eliminated from the analysis. N-J phylogenetic trees were constructed with the Maximum Composite Likelihood method (ML). The names of the isolates from the present study are in bold in the trees. Maximum likelihood bootstrap support values are given at the nodes, respectively.

## 5. Conclusions

This research identified *B. dothidea*, *T. roseum*, *A. alternata*, and *P. kenyana* as the primary fungi causing ring rot, brown spot, black spot, and soft rot in loquat fruit in Yunnan, China. The varying virulence between species and strains highlights the complexity of postharvest fruit rot dynamics, indicating that closely related isolates exhibit different levels of pathogenicity. The virulence varied with species, even strains of the same species. The incidence rates of 4%, 6%, 6%, and 12% for the respective rot types underscore a significant concern for loquat production in the region. The findings contribute to the epidemiological understanding of these pathogens and serve as a reminder for postharvest management practices. Effective control measures are crucial in mitigating the impact of these diseases, which can result in substantial economic losses for farmers and compromise fruit quality. This study is the first to show that *B. dothidea*, *T. roseum*, and *P. kenyana* are involved in postharvest rot of loquats worldwide, and it is also the first evidence that *A. alternata* is responsible for the black spots of loquat fruits in Yunnan. The findings gained from this study are crucial for epidemiology and provide a cautionary tale for postharvest management of loquat in Yunnan, China. Further research is warranted to explore the ecological roles of these pathogens and develop targeted management strategies for agricultural practices. Understanding the interactions between these fungi and their environment will be crucial for formulating effective control measures and ensuring the sustainability of loquat cultivation, both in Yunnan specifically and globally.

## Figures and Tables

**Table 1:** List of fungi that cause loquat fruit decay.

No.	Pathogens	Period	Location	Authors and References
1	*Colletotrichum gloeosporioides*	Before-harvest	Taxila and Wah Cantt, Pakistan	Naz, F. [19]
		Postharvest	Alacant Province, Spain	Palou, L. [5]
		Postharvest	Nagasaki Prefecture, Japan	Takata, Y. [6]
2	*C. godetiae*	Before-harvest	Tetela, Morelos State, Mexico	Juárez-Vázquez, S.B. [20]
3	*C. acutatum*	Postharvest	Fujian Province, China	Gu, H. [7]
		Postharvest	Jiangsu Province, China	Cao, S. [8]
4	*C. fioriniae*	Postharvest	Nagasaki Prefecture, Japan	Takata, Y. [6]
		Before-harvest	Nagasaki Prefecture, Japan	Poti, T. [22]
5	*C. scovillei*	Postharvest	Zhejiang Province, China	Wu, J. [9]
6	*C. eriobotryae*	Before-harvest	Taiwan Province, China	Damm, U. [21]
7	*C. nymphaeae*	Postharvest	Nagasaki Prefecture, Japan	Takata, Y. [6]
		Before-harvest	Nagasaki Prefecture, Japan	Poti, T. [22]
		Before-harvest	Taiwan Province, China	Damm, U. [21]
		Postharvest	Sichuan Province, China	Wu, W.X. [10]
8	*Pestalotiopsis eriobotryfolia*	Postharvest	Fujian Province, China	Gu, H. [7]
9	*P. sensu*	Postharvest	Alacant Province, Spain	Nozawa, S. [11]
10	*P. guepini*	Before-harvest	Buenos Aires, Argentina	Perelló A.E. [23]
	*P. theae*	Before-harvest	Anhui Province, China	Chen, Y. [24]
11	*Neopestalotiopsis clavispora*	Postharvest	Alacant Province, Spain	Palou, L. [5]
		Postharvest	Pakistan	Abbas, M.F. [12]
		Postharvest	Spain	Palou, L. [13]
12	*Alternaria alternata*	Before-harvest	Thessaloniki, Greece	Tziros, G.T. [25]
		Before-harvest	Palestine	Batta, Y. [26]
		Postharvest	Islamabad, Pakistan	Bibi, H. [14]
13	*A. tenuissima*	Postharvest	Fujian Province, China	Gu, H. [7]
14	*Alternaria* sp.	Before-harvest	Taiwan Province, China	Ko, Y. [27]
15	*Fusicladium eriobotryae*	Before-harvest	Spain	González-Domínguez, E. [28]
16	*Botrytis cinerea*	Postharvest	Alacant Province, Spain	Palou, L. [5]
17	*Diplodia seriata*	Postharvest	Alacant Province, Spain	Palou, L. [5]
		Before-harvest	Alicante Province, Spain	Palou, L. [29]
		Before-harvest	Punjab and Khyber Paktoon Khawa, Pakistan	Abbas, M.F. [30]
18	*Penicillium expansum*	Postharvest	Alacant Province, Spain	Palou, L. [5]
19	*Rhizopus stolonifer*	Postharvest	Alacant Province, Spain	Palou, L. [5]
		Postharvest	Rawalpindi and Swat, Pakistan	Aslam, M.F. [15]
20	*Ceratobasidium* sp.	Postharvest	Guangdong Province, China	Li, S.N. [16]
21	*Fusarium oxysporum*	Before-harvest	Islamabad, Pakistan	Niazi, F. [31]
22	*F. solani*	Before-harvest	Punjab Province, Pakistan	Abbas, M.F [32]
23	*Geotrichum candidum*	Postharvest	Lahore, Pakistan	Hafeez, R. [17]
24	*Neofusicoccum parvum*	Before-harvest	Chongqing, China	Zhai, L. [33]
25	*Diplocarpon mespili*	Before and Postharvest	Spain	Gariglio, N. [18]
26	*Monilinia fructicola*	Before-harvest	Wuhan, Hubei Province, China	Yin, L. [34]

**Table 2:** Morphological characteristics of the fungal species causing loquat postharvest fruit rot in China.

Morphotype Group	Strain Name	**Colony Character on PDA**	Conidia	Similar Species
Group Ⅰ *Botryosphaeria dothidea*	GJW41-13 GJW42-17	Circular and initially white colonies gradually turned gray-green, with a dark green color on the back, and featured short and thick aerial hyphae with an irregular colony margin. Colony diameters were 8.0 cm and 9.0 cm after 3 d at 28 °C in the light, respectively.	Hyaline and subcylindrical, aseptate, 17.5–24.1 × 5.7 to 7.2 μm, L/W = 2.7–4.1 (mean 21.0 × 6.6 μm, average L/W = 3.2, *n* = 30)	*Botryosphaeria dothidea* [(20-)23-27(-30) × 4-5(-6) (mean 26.2 × 5.4 μm)]
Group Ⅱ *Trichothecium roseum*	GJW43-2 GJW43-10 GJW43-17	Circular and initially white colonies gradually produced dense, pink, and circular structures (conidiophores and conidia) with a rough colony margin. Colony diameters were 2.7 cm, 2.7 cm, and 2.8 cm after 3 d at 28 °C in the light, respectively.	Produced in clusters, smooth, hyaline, thick-walled, 1-septa, ellipsoid to pyriform, 9.1–16.3 × 4.5–8.8 μm, L/W = 1.4–2.6 (mean 12.8 × 6.3 μm, average L/W = 2.1, *n* = 30)	*Trichothecium roseum* (10 to 18 × 7 to 9.5 μm)
Group Ⅲ *Alternaria alternata*	GJW44-5 GJW44-16 GJW46-2	Initially a white colony and turned olive green to black 7 days post-incubation. Featured pale brown, thick, and cottony aerial hyphae, with a reserve of black surrounded by a light-brown circle. Colony diameters were 6.7 cm, 5.6 cm, and 7.2 cm after 3 d at 28 °C in the light, respectively	Produce chained conidia singly, separated, and pale brown conidiophores. Conidia yellow-brown or black-brown, obclavate, subglobose, ellipsoid, with 1–5 transverse septa and 1–3 longitudinal septa, 13.4–42.9 × 7.7–22.0 µm, L/W = 1.5–4.6 (mean 27.6 × 13.5 µm, average L/W = 2.8, *n* = 30)	*Alternaria alternata* [26–30 × 5–9 µm with 4–7 transverse septa and a few or no longisepta]
Group Ⅳ *Pestalotiopsis kenyana*	GJW67-9 GJW67-12	Smooth-edged, dense, whitish, with sparse aerial mycelium on the surface, produced a yellowish or black oil-drop mass (conidiophores and conidia) and a yellowish reserve with an irregular margin—colony diameter was 3.3 cm after 4 d at 28 °C in the light.	Fusoid, ellipsoid, straight or slightly curved, 4-septate, 20.9–27.6 × 4.0–5.4 μm, L/W = 4.5–6.9 (mean 24.3 × 4.6 μm, average L/W = 5.5, *n* = 20); three median cells were darker than other cells; apical cell with 2–3 tubular appendages (mainly three) ranging from 6.6 to 16.3 μm, basal cell with one tubular appendage ranging from 4.1 to 7.6 μm.	*Pestalotiopsis kenyana* [17.5–22 × 6–7 µm (mean 20 × 6.5 µm)]

Note: L/W = length-to-width ratio.

**Table 3:** Disease incidence and disease index of *Alternaria alternata* GJW44-5, GJW44-16, and GJW46-2 at a relative humidity of 50%**.**

Temperature/℃	*Alternaria alternata* GJW44-5		*A. alternata* GJW44-16		*A. alternata* GJW46-2	
	Disease Incidence/%	Disease Index	Disease Incidence/%	Disease Index	Disease Incidence/%	Disease Index
15	100	32	100	46	100	62
25	100	42	100	62	100	70
30	100	68	100	70	100	82

**Table 4:** Disease incidence and disease index of *Alternaria alternata* GJW44-5, GJW44-16, and GJW46-2 at 25 °C.

Humidity/%	*Alternaria alternata* GJW44-5		*A. alternata* GJW44-16		*A. alternata* GJW46-2	
	**Disease Incidence/%**	**Disease Index**	**Disease Incidence/%**	**Disease Index**	**Disease Incidence/%**	**Disease Index**
45	90	32	90	32	90	36
55	100	34	100	40	90	36
65	100	40	100	40	100	38
75	100	44	100	40	100	38
85	100	44	100	44	100	44
95	100	44	100	42	90	38

**Table 5:** PCR reaction primers (forward and reverse) and protocols for amplification of gene loci of each fungal genus.

Locus	Primer Names and Primer Sequences	PCR Protocols	References
ITS	ITS1: TCC GTA GGT GAA CCT GCG G ITS4: TCC TCC GCT TAT TGA TAT GC	(95 °C 30 s, 55 °C 50 s, 72 °C 1 min) × 39 cycles	White, T.J. [71] Udayanga, D. [75]
LSU	LROR: ACC CGC TGA ACT TAA GC LR5: ATC CTG AGG GAA ACT TC	(95 °C 1 min, 55 °C 2 min, 72 °C 90 s) × 35 cycles	Vilgalys, R. [72] Doilom, M. [74]
TEF	EF1-728F: CAT CGA GAA GTT CGA GAA GG EF1-986R: TAC TTG AAG GAA CCC TTA CC	(95 °C 30 s, 58 °C 50 s, 72 °C 1 min) × 39 cycles	Vilgalys, R. [72] Udayanga, D. [75]
TUB	T1: AAC ATG CGT GAG ATT GTA AGT Bt2b: ACC CTC AGT GTA GTG ACC CTT GGC	(95 °C 30 s, 58 °C 50 s, 72 °C 1 min) × 39 cycles	Jiang, N. [73] Udayanga, D. [75]
RPB2	RPB2-5F2: GGG GWG AYC AGA AGA AGG C fRPB2-7CR: CCC ATR GCT TGY TTR CCC AT	(95 °C 1 min, 52 °C 2 min, 72 °C 90 s) × 35 cycles	Jiang, N. [73] Doilom, M. [74]

## Data Availability

The sequences of GJW41-13, GJW42-17, GJW43-2, GJW43-10, GJW43-17, GJW44-5, GJW44-16, GJW46-2, GJW67-9, and GJW67-12 (PV017472~PV017481, PV017485~PV017486 for ITS, PV069446~PV069448 for GAPDH, PV069450~PV069452 for TUB2), and the ACT gene of GJW200-1 (PV069453), GJW200-2 (PV069454), and GJW202-2 (PV069449) have been submitted to NCBI and opened to the public.
